# Overlap in Erotic Age Preferences: Support for the Chronophilia Theory in a Community Self-Report Sample of Males

**DOI:** 10.5964/sotrap.13135

**Published:** 2025-06-12

**Authors:** Valerie Arenzon, Ashley Curtis, Hannah de Almeida, Crystal Evanoff

**Affiliations:** 1 Department of Psychology, Université de Montréal, Montreal, Quebec, Canada; 2 Department of Psychology, University of Canterbury, Christchurch, New Zealand; 3 Department of Psychology, Carleton University, Ottawa, Ontario, Canada; 4 Department of Psychology, University of British Columbia Okanagan, Kelowna, British Columbia, Canada; University of Lincoln, Lincoln, United Kingdom

**Keywords:** chronophilia, pedophilia, hebephilia, sexual age orientation

## Abstract

Seto (2017) proposes sexual orientation not only varies as a function of gender, but also as a function of age. Few studies have examined the conceptualization of sexual age orientation. The current study evaluates the polymorphism of sexual interest in children (i.e., simultaneous attraction to multiple age categories), the exclusivity of sexual interest (i.e., attraction to children only or children *and* adults), and gender preference (i.e., preference for males or females) in the general population. Data were obtained through adult males (*N* = 170) using online self-reports (overlapping sample from Mundy & Cioe, 2019). Findings revealed that most individuals reporting sexual attraction to multiple age categories had a higher degree of preference for one age group over others. When looking at sexual interest in children among teleiophilic individuals, the concordance indices were higher between adjacent age groups compared to distant age groups. Specifically, among individuals who reported teleiophilic sexual interest, 36% were also reporting hebephilic sexual interest (adjacent category), while 17.7% reported pedophilic sexual interest (non-adjacent category). Finally, there was a significant positive correlation between pedophilic interest and hebephilic interest (τ_b_ = .602, *p* < .001). Together, results provide support for the chronophilia theory. Concordance indices in future studies may inform differences between exclusive and nonexclusive interest in children and aid the development of informed risk assessment tools and destigmatized prevention programs.

## Highlights

- Males have concordant sexual interests to multiple age groups.
- Most males with sexual interests in children also have sexual interests in adults.
- Concordant sexual interest is more likely to occur between adjacent age boundaries.
- Overlap in sexual interests across age groups differs based on gender preferences.

Sexual interests in modern society are diverse. A growing body of literature on paraphilias (i.e., atypical sexual interests) find a minority of people with pedophilic (i.e., sexual interest for pre-pubescent children) and hebephilic (i.e., sexual interest for pubescent children) interests (Gerwinn et al., 2018; Stevens & Wood, 2019). The true prevalence of sexual interest in children among the general population remains difficult to assess due to variability of estimates across studies. A recent systematic review by Savoie et al. (2021) found the mean prevalence rate for sexual interest in pre-pubescent children was 2% (*n* = 9 studies), and 3% for interest in pubescent children (*n* = 3 studies). To date, little is known about sexual attraction patterns of pedophilic and hebephilic individuals in the general population, with most of our understanding stemming from individuals (mostly men) adjudicated for sexual offences against children (Seto, 2018). Specifically, it is currently unclear if pedophilia and hebephilia represent exclusive dichotomous taxons or if sexual attraction to children can be understood as a spectrum alongside an age/maturity gradient. As such, the current study aims to explore the patterns of sexual attraction to children in a self-report community sample of males.

A substantial amount of empirical work has been dedicated to understanding whether sexual interest in children would be better conceptualized as taxons (i.e., separate categories; e.g., Hanson, 2010; McPhail et al., 2018) or represented dimensionally on a gradient (Bailey et al., 2016; Seto, 2012, 2017). Akin to how gender preferences (e.g., straight, gay) are largely stable across the lifespan (Mock & Eibach, 2012; Savin-Williams et al., 2012), sexual interest in children is also reported as being stable across the life span (Grundmann et al., 2016). In a two-part study of self-identifying pedophiles seeking help through the *Prevention Project Dunkelfeld* (*N* = 494), researchers found that most men (between 58% and 72%) experienced a pubertal onset of their pedophilic/hebephilic sexual arousal (Grundmann et al., 2016). A subsequent assessment of stability and variability of sexual arousal (*N* = 121) over an extended period (i.e., average of 29 months) showed either no change or minimal change (i.e., ± 1 within the response categories) in sexual arousal. Tozdan and Briken (2015) also found individuals who acknowledged their sexual interest in children at an earlier age experienced less change in interest over time.

The prevailing evidence indicating both the early onset of sexual interest in children (e.g., Grundmann et al., 2016) and its enduring stability throughout an individual's life (Tozdan & Briken, 2015) has prompted numerous researchers to predict a taxonic structure of sexual interest in children (Cantor & McPhail, 2015; Hanson, 2010; McPhail et al., 2018; Schmidt et al., 2013). Studies employing methods such as phallometric testing (e.g., Cantor & McPhail, 2015) and taxometric analyses (e.g., Schmidt et al., 2013) have found evidence for clear boundaries between different types of sexual interest, including exclusively pedophilic and non-exclusively pedophilic groups. These findings suggest sexual interest in children can be understood as comprising discrete categories rather than existing on a continuum.

However, research has also provided evidence that sexual interest in children is dimensional rather than a taxonic structure (Blanchard et al., 2012; Stephens et al., 2017), suggesting that pedophilia would be better understood as a dimensional structure of sexual interest in children. The divergent findings between studies supporting the taxonic structure versus the dimensional structure of pedophilia may be partially explained by their respective samples. The studies supporting a taxonic structure of pedophilia largely use sample of males who committed sexual offenses against children, which can be viewed as the maladaptive end of a spectrum; therefore, their findings may not be transposed to non-offending individuals with sexual interests in children (Stephens et al., 2017). Research conducted among community samples may favor a multidimensional conceptualization of a sexual age orientation.

## The Chronophilia Theory

**Figure 1:**
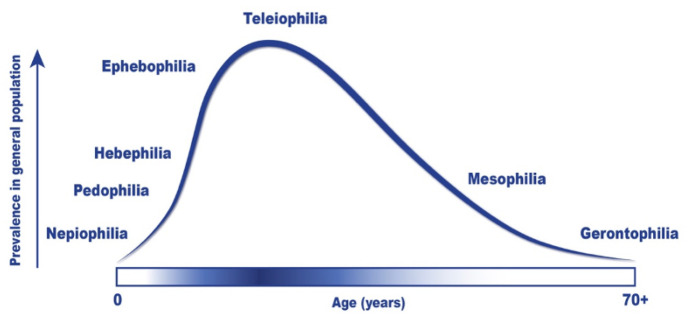
Theoretical Distribution of the Male Chronophilia in the General Population *Note.* The relative frequency of chronophilias in men is unknown.

The chronophilia theory (Seto, 2017) posits that sexual orientation should be expanded to include other factors beyond gender, such as age. According to this theory, individuals can hold various (and simultaneous) age preferences. The first mention of a sexual age orientation was captured by the chronophilia theory [chrono (time) + philia (love)] proposed by John Money in 1986. The most recent consideration for the chronophilia theory is Seto’s (2017) discussion of the multi-dimensional framework of *chronophilias* (i.e., sexual age preferences). Specifically, Seto (2017) suggested that erotic age preferences should be considered as a form of sexual orientation, ranging from *nepiophilia* (i.e., attraction to infants) to *gerontophilia* (i.e., attraction to elderly individuals). Accordingly, *chronophilias* would be represented by an ordered, continuous, and unimodal distribution of age preferences (see Figure 1). Figure 1 represents a theoretical distribution of male chronophilia in the general population, with teleiophilia being the most prevalent. The distribution shows that a larger portion of males have a sexual preference for minors than for the elderly, thus resulting in a positively skewed curve with a steeper peak on the left and a long right tail. The lack of knowledge regarding female chronophilia prevents the generalization of Seto’s distribution to all sexes. However, according to Seto (2017), the lack of data on female chronophilias suggests the prevalence would be much lower and show a negative (rather than positive) distribution.

### Non-Exclusive Sexual Interests Across Age Categories

The chronophilia theory proposes a nuanced view of sexual orientation, suggesting sexual interest spans across an age spectrum in a manner similar to the gender orientation spectrum. Akin to the idea that gender orientation can include sexual attraction to multiple genders simultaneously (i.e., bisexuality), Seto (2017) introduced the idea that sexual attractions can bridge different age groups simultaneously (i.e., nepiophilia, pedophilia, hebephilia, teleiophilia, gerontophilia). Consequently, the chronophilia theory challenges the traditional view that erotic age interest is confined to one age category. Existing research has already offered evidence of non-exclusivity of sexual interests across age categories. For example, a study conducted by Beier and colleagues (2009) with a sample of 215 pedophilic individuals seeking mental health services found 39% had non-exclusive sexual interests, indicating their sexual attraction spanned more than one age category. Although the true estimates of chronophilias in the community are unknown, Seto (2017) argues the non-exclusivity of sexual age orientation exists on both a population and an individual level.

### Adjacent Age Interests

The stimulus generalization gradient proposed by Blanchard et al. (2012) provides a theoretical foundation for understanding how sexual preferences might extend beyond exclusive taxonic categories. The stimulus generalization gradient suggests individuals are likely to have a predominant sexual interest for a specific age group alongside descending sexual interests for adjacent age groups. For example, sexual attraction to mature adult women is more likely to co-occur with a sexual interest in pubescent girls rather than with infants because of the developmental proximity. Similarly, it would be unlikely for an individual to have a sexual interest for both prepubescent children and the elderly (i.e., gerontophilia). As such, this model suggests a gradient of attraction that diminishes as the developmental difference from the primary age interest increases.

Blanchard et al. (2009) evaluated absolute ascertainment (i.e., intense sexual interest in children) and relative ascertainment (i.e., sexual interest for children is greater than sexual interest in adults), in a sample of *N* = 402 heterosexual men. The study found 130 teleiophilic heterosexual men (i.e., high penile responses to adult women) within the sample were also aroused by prepubescent girls. In contrast, pedophilic men (*n* = 272) demonstrated a weak penile response to prepubescent girls, and an even weaker response to adult women. Similarly, another study found that teleiophilic heterosexual men showed mild physiological arousal to pubescent and prepubescent girls (Lykins et al., 2010). These findings support the chronophilia theory’s claim that individuals have sexual interest outside their preferred age category; however, it does not provide insight into the patterns of overlap in adjacent-age categories in community samples.

Furthermore, a study looking at sexual attraction to children via an online survey of 306 men found individuals report at least some attraction to age categories adjacent to their primary attraction (Martijn et al., 2020). Specifically, 73% reported attraction to polymorphic maturity categories (i.e., multiple age categories), and 79% had non-exclusive sexual interests (i.e., attraction to children and adults). Although the study focused on romantic attractions, findings support the multidimensional sexual age orientation theory.

### Interplay Between Age Orientation and Other Dimensions of Sexual Attraction

Seto (2017) also anticipated complex interactions between sexual age attraction and different dimensions of sexual attraction such as gender and other paraphilias. Blanchard and colleagues (2012) explored the complex nature of sexual attraction by comparing two distinct models: one focusing on the level of attraction to specific traits (e.g., age, gender), and the other focusing on the level of attraction to global morphological similarities of individuals. Phallometric measures conducted on a sample of 2,278 men suggested men react sexually by regarding other humans as points along a single bipolar dimension of physical similarity (like the Kinsey Scale of erotic age preference; Kinsey, Pomeroy, & Martin, 1948); this dimension positions adult men at one end, children who have not reached puberty near the center, and adult women at the opposite end. The results provide evidence for a continuum of sexual attraction that incorporates both specific traits like age and gender, as well as more global morphological similarities. In this way, individuals’ sexual preferences might span a wide range of ages and morphologies, underscoring the multifaceted nature of sexual attraction.

Further investigations into the combination of age and gender factors in erotic preferences provided additional evidence for a spectrum of sexual age orientation (Bailey et al., 2016). In their study examining sexual offending against children in a sample of 1,102 men from online forums dedicated to sexual interest in children, Bailey et al. (2016) found that self-reported sexual interests were strongest for the preferred gender/age category, and next strongest for adjacent gender/age categories, consistent with the stimulus generalization gradient. This pattern reinforces the idea that sexual interest is not confined to rigid categories but may instead flow across a gradient of sexual attraction as proposed by Blanchard et al. (2012). However, although their study used a non-forensic sample, their participants were recruited from specific forums for sexual interest in children, which limits the generalizability of their findings to the general population. Beyond these studies, evidence regarding patterns of overlapping erotic age preferences and gender preference in community samples remains sparse. In a subsequent study using multidimensional scaling across four datasets (mostly comprised of men attracted to children and men with histories of offending against children), Bailey et al. (2021) found evidence supporting the sex/age gradient. Precisely, sex and sexual maturity significantly shape the level of sexual interest.

## Current Study

The current study sought to evaluate the concept of sexual age orientation within Seto’s (2017) chronophilia framework. Specifically, the investigation centered around three main assumptions of the chronophilia theory. First, we hypothesized that individuals would exhibit a pattern of sexual age attraction that was predominantly non-exclusive. Specifically, we predicted that individuals would hold simultaneous sexual interests among various age categories, supporting Seto’s (2017) idea that sexual age orientation exists on a fluid, non-exclusive spectrum. Second, in alignment with the sexual response gradient proposed by Seto and colleagues (1999), we hypothesized that there would be higher concordance (i.e., greater overlap) in sexual interest between adjacent age categories (e.g., hebephilia and teleiophilia) compared to non-adjacent age categories (e.g., pedophilia and teleiophilia). Lastly, Seto expected the age orientation to interact with other dimensions such as the gender orientation. As such, our study examined whether patterns of sexual interest remained consistent across different genders of interest (i.e., interest in males vs. females). We hypothesized that there would be no differences in concordance between sexual interest in males and females. To test these hypotheses, we calculated concordance indices alongside correlational analyses of self-reported sexual interests among a community sample of males.

## Materials and Method

### Participants

The current study used an overlapping sample from an online self-report survey gathered by Mundy and Cioe (2019). A total of 529 participants were recruited from either a Canadian university using a psychology class subject pool (*n* = 259), or from online sexual forums on Reddit (*n* = 270). The primary author of Mundy and Cioe (2019) contacted leading moderators of Reddit forums (r/sex, r/SampleSize, r/TwoXChromosomes) to explain the study and gain consent to post an ad-poster and link to the online questionnaire. The two sexual forums (i.e., r/sex and r/TwoXChromosomes) are subreddit forums designed for general discussions pertaining to sex education and advice; neither forums are geared toward individuals with sexual interest in children and are moderated to ensure appropriate behaviour. The purpose of the present analyses required a sample of male respondents that answered all questions about sexual interests in children and adults. Hence, the final sample included 170 adult males (*n* = 43 university students, *n* = 127 online sample) between age 17 and 64 (*M_age_* = 27.0). Due to length of survey, a single question was used to self-report honesty. In prior studies, the single-question approach has yielded useful information within the context of sensitive topics such as sexual behaviour (e.g., Zimmerman & Langer, 1995). Hence, participants included in the analysis had to complete all questions relevant to the following analyses and rate their honesty in the survey as a 4 (*Mostly Honest*) or 5 (*Completely Honest*). Individuals who did not meet these criteria (*n* = 0) were removed from the current analysis. An attention check item was also included in the questionnaire to ensure data quality and validity of responses.

### Measures

#### Sexual Life and Sexual Behaviour Questionnaire (SLSB)

The current study used the Sexual Life and Sexual Behaviour Questionnaire (SLSB), a standardized survey tool inquiring about sexual health. Translated from German, the SLSB is a revised version of the Questionnaire on Sexual Experiences and Behaviour (Fragebogen zum Sexuellen Erleben und Verhalten) developed at the Institute of Sexology and Sexual Medicine of a medical university in Berlin (Ahlers et al., 2011). The content of the questionnaire is divided into multiple sections: (1) demographic information, (2) sexual and gender identity, (3) sexual tendencies, (4) views and experiences, (5) and medical illnesses and procedures. The current study only used the sections for demographic information, and self-reported sexual tendencies. Notably, to assess sexual orientation, participants were asked “What is your sexual orientation?”, where they selected from a 7-point scale including “exclusively women”, “mostly women”, “both men and women”, “mostly men”, “exclusively men”, “asexual” or “other”.

##### Sexual Tendencies

Sexual tendencies, comprised of several questions on paraphilic interests and associated sexual behaviours, were the focus of the current study. Sexual interests were assessed through questions differentiating between fantasy and fantasy-related behaviours (e.g., masturbation). Importantly, the questions did not use age or diagnostic terminology when describing a person. For example, in the first question, participants were asked “*How sexually arousing do you find young boys and the prepubescent male body (without pubic hair)?*”. To answer, participants were presented with a 5-point Likert scale including “*Not at All*”, “*Slightly*”, “*Moderately”*, “*Very*”, and “*Extremely*”. If participants reported any level of sexual interest beyond *Not at All*, they were prompted to answer additional questions regarding fantasy, behaviour, and distress. In the second question, participants were asked how long they had the interest. Paraphilia-associated sexual behaviours were then evaluated by asking questions about general fantasies (e.g., “How often do you engage in this fantasy during sexual fantasies?), masturbation fantasies (e.g., “How often do you engage in this fantasy during masturbation fantasies?”), and sexual behaviours (e.g., “How often do you engage in this fantasy during sexual behaviour?”) using a 5-point numeric scale ranging from 1 (Never) to 5 (Almost Always). Distress was evaluated in the remaining two questions, including: (1) how often the participant was negatively impacted by the fantasy, and (2) how often the fantasy negatively impacted their relationships. Responses were scored on a 5-point scale ranging from 1 (Not at all) to 5 (Extremely).

#### Combination of Sexual Interests Scores

To assess sexual interest in children and adults regardless of gender preferences, combined variables were created using the largest score of the two subordinate variables. Specifically, sexual interests in prepubescent females and prepubescent males were combined into an overall interest in prepubescent children using the largest score of the two variables. Sexual interests in pubescent females and pubescent males were combined into an overall interest in pubescent children using the largest score of the two variables. Finally, sexual interests in adult females and adult males were combined into an overall interest in adults using the largest score of the two variables.

#### Categorization of Sexual Interests

Further analyses of overlap between sexual interests required the creation of dichotomous variables (i.e., sexual interest present, sexual interest absent). Due to the nature of the planned analyses (see below; Joyal & Carpentier, 2022), sexual interests were categorised as present if participants rated their interests as *slightly, moderately, very,* or *extremely*. Accordingly, sexual interests were categorised as absent when participants rated their sexual interests as *not at all*. Participants were classified as having a pedophilic interest (i.e., self-reported any level of sexual interest in prepubescent children), hebephilic interest (i.e., self-reported any level of sexual interest in pubescent children), and teleiophilic interest (i.e., self-reported any level of sexual interest in mature adults).

### Procedure

#### Recruitment

To recruit online participants, researchers contacted moderators of forums to post a recruitment ad-poster and survey link for the study on Reddit (Mundy & Cioe, 2019). A debriefing form was also included at the end of the questionnaire. Finally, participants received a separate link (i.e., independent of questionnaire answers) to provide their contact information for a gift card draw. For students in the subject pool, the separate link was used to attribute extra course credit for completing the questionnaire.

Although the current study did not pose risks for participants beyond their risk of daily lives, potential disclosure of highly sensitive information required extensive protection of participants’ identities. Hence, no personal information was requested, and IP addresses were deleted upon data retrieval. Participants were informed that no penalty would be imposed if they decided to withdraw from the study. REB approval was granted for the original study (H15-00752). As the dataset used in the current study involves a secondary analysis of anonymous data, no additional REB approval was required.

### Data Analysis

Concordance indices were developed to account for base rates and measure overlap between sexual interests in subgroups of individuals with chronophilic interests. The concordance index is calculated by dividing the number of concordant pairs by the sum of concordant and discordant pairs. For example, when an individual with a sexual interest in prepubescent children had also a sexual interest in pubescent children, then his interests represented a *concordant pair*. In contrast, when an individual with a sexual interest in prepubescent children did not have a sexual interest in pubescent children, then his interest represented a *discordant pair*. Concordance is a useful metric in this study because it indirectly accounts for base rates of sexual interest in this sample. Rather than just capturing the raw total number of individuals who report simultaneous interests (e.g., Teleiphilia-Hebephilia), concordance considers the number of individuals with one interest (e.g., Hebephilia) *relative* to the total proportion of individuals with another interest (e.g., Teleiophilia). As such, concordance contextualizes the overlap of sexual interests by accounting for the prevalence of each type of sexual interest in the overall sample.

It is worthwhile noting that the number of pedophilic-hebephilic concordant pairs may differ from the hebephilic-pedophilic concordant pairs, as not all individuals within the hebephilic group may have a sexual interest for pedophilia. The concordance index goes beyond simple overlap as it provides insight into sexual interests within each group of chronophilias (How many individuals who reported sexual interest in prepubescent children also reported sexual interest in pubescent children? How many individuals who reported a sexual interest in pubescent children also reported a sexual interest in prepubescent children?).

Kendall’s Tau-b, a nonparametric correlation test, was conducted to examine the similarity between the rank order of one sexual interest rating (i.e., *not at all*) to the rank order of another sexual interest rating (i.e., *extremely*). Kendall’s Tau-b was chosen over Pearson’s *r* because it is more appropriate for ordinal data and does not assume a normal distribution. Specifically, the purpose of the correlational analyses was to determine the level of interest in one age category (e.g., pedophilia) to another age category (e.g., teleiophilia). An alpha value of .05 was used as an indicator of statistical significance throughout analyses. Measures of effect size were also considered in the interpretation and the discussion of results. A correlation of τ_b_ = .10 was considered small, τ_b_ = .30 as medium, and τ_b_ = .50 and larger as large (Cohen, 1988). Given our sample size, we prioritized assessing the prevalence of effect sizes over p values when interpreting findings.

## Results

### Prevalence of Chronophilias

Prevalence rates of sexual interest in children and in adults were provided for the overall sample. The participant's self-reported gender identity, sexual orientation, and mean age were separated by chronophilic interests (see Table 1). Further breakdown of sexual interest by gender preference found differences in prevalence rates between genders. About 2 out of 10 (17.6%; *n* = 30) reported some level of pedophilic interest for females, while 6.5% (*n* = 11) reported some level of pedophilic interest for males. Moreover, a total of 35.3% (*n* = 60) of participants reported some level of hebephilic interest for females, and 7.1% (*n* = 12) reported some level of hebephilic interest for males. Finally, 92.4% (*n* = 157) reported some level of teleiophilic interest for females, and 35.3% (*n* = 60) reported some level of teleiophilic interest for males.

**Table 1:** Descriptive Statistics for Age, Gender, and Sexual Orientation per Chronophilia

Characteristics	Entire Sample (*n* = 170)	Pedophilic Interest (*n* = 31)	Hebephilic Interest (*n* = 60)	Teleiophilic Interest (*n* = 164)
Age (years)	26.97 (*SD* = 9.5)	25.1 (*SD* = 6.77)	25.3 (*SD* = 8.07)	27.2 (*SD* = 9.57)
Gender				
Male	96.5	83.9	76.7	81.7
Female	2.1	3.2	3.3	1.8
Other	1.4	3.2	1.7	0.6
Missing	–	9.7	13.6	15.9
Sexual Orientation				
Exclusively Women	67.5	51.6	56.7	67.7
Mostly Women	18.3	25.8	28.3	18.3
Men and Women	7.7	12.9	11.7	7.9
Mostly Men	1.8	–	–	1.2
Exclusively Men	4.1	6.5	1.7	3.7
Asexual	0.6	–	–	0.6
Missing	0.6	3.2	1.7	0.6

Additionally, frequencies of ratings of sexual interests for each chronophilia per gender preference are shown in Table 2. The mean rating for *pedophilia – female* (*M* = 1.32, *SD* = .80) was lower than both *hebephilia – female* (*M* = 1.65, *SD* = .48) and *teleiophilia – female* (*M* = 4.31, *SD* = 1.16). For sexual interests in males, the mean rating for *pedophilia – male* (*M* = 1.11, *SD* = 0.475) was close to the mean rating for *hebephilia – male* (*M* = 1.08, *SD* = .29), and lower than *teleiophilia – male (M* = 1.64, *SD* = 1.10). When looking at the maximal score of sexual interests regardless of gender preference, the mean rating for *pedophilia* (*M* = 1.34, *SD* = .82) was lower than both *hebephilia* (*M* = 1.65, *SD* = 1.06) and *teleiophilia* (*M* = 4.47, *SD* = .94).

**Table 2:** Frequencies of Ratings of Sexual Interests per Gender Preferences

Sexual Interest	Gender	Not at all	Slightly	Moderately	Very	Extremely
		*n* (%)	*n* (%)	*n* (%)	*n* (%)	*n* (%)
Pedophilia	Female	140 (82.4%)	14 (8.2%)	8 (4.7%)	7 (4.1%)	1 (0.6%)
Hebephilia	Female	110 (64.7%)	29 (17.1%)	16 (9.4%)	10 (5.9%)	5 (2.9%)
Teleiophilia	Female	13 (7.6%)	4 (2.4%)	5 (2.9%)	44 (25.9%)	104 (61.2%)
Pedophilia	Male	159 (93.5%)	7 (4.1%)	2 (1.2%)	1 (0.6%)	1 (0.6%)
Hebephilia	Male	158 (92.9%)	11 (6.5%)	1 (0.6%)	–	–
Teleiophilia	Male	110 (64.7%)	24 (14.1%)	23 (13.5%)	6 (3.5%)	7 (4.1%)

Among participants who reported multiple sexual interests for females (*n* = 60), 86.67% (*n* = 52) reported having a higher sexual interest for one chronophilia over the other two chronophilias. Of these participants, 49 had a dominant sexual interest for teleiophilia and three had a dominant sexual interest for hebephilia. Moreover, seven participants had two dominant sexual interests, six of which had dominant sexual interests for hebephilia and teleiophilia, while one had pedophilia and teleiophilia. Finally, one participant had equal level of sexual interest across the three chronophilias.

Among participants who reported multiple sexual interests for males (*n* = 11), 72.73% (*n* = 8) reported having a higher sexual interest for one chronophilia over the other two chronophilias, all of which were for teleiophilia. One participant reported having two equally dominant sexual interests, which were pedophilia and teleiophilia. Finally, two participants had equal sexual interests between two chronophilias and no other interest.

When looking at the combined ratings of sexual interests (i.e., regardless of gender preference), a total of 35.88% participants had multiple sexual interests (*n* = 61). Among participants with multiple sexual interests, 85.25% (*n* = 52) reported having a higher sexual interest for one chronophilia over the other two chronophilias. Of these, 49 had a dominant sexual interest for teleophilia and three had a dominant sexual interest for hebephilia. Moreover, eight participants had two dominant sexual interests, six of which had dominant sexual interests for hebephilia and teleiophilia, while two had equal pedophilia and teleiophilia interests. Finally, one participants had equal sexual interests between hebephilia and teleiophilia and no interest in pedophilia.

### Concordance Indices

Concordance indices were computed to examine overlapping sexual interests among participants reporting a chronophilic interest, with higher concordance indices indicating greater overlap in attraction between the two age groups. As shown in Table 3, concordance indices between adjacent erotic-age preference groups were higher compared to distant erotic-age preference groups. When looking at sexual interests regardless of gender, most participants who reported a sexual interest for pedophilia also reported a sexual interest for hebephilia (93.5%). Approximately half (48.3%) of those who reported sexual interest for hebephilia also reported a sexual interest for pedophilia. Overall, concordance indices for participants with chronophilic interests show overlap between erotic-age preferences groups. Therefore, for most participants, chronophilic sexual interests are nonexclusive.

**Table 3:** Concordance Indices for Participants With Chronophilic Interests

Pairs of Sexual Interest		Concordance Indices		
		Gender		
		Combined (Any Gender)	Female	Male
		%	%	%
Pedophilia	Hebephilia	93.5	93.3	72.7
	Teleiophilia	93.5	93.3	81.8
Hebephilia	Pedophilia	48.3	46.7	66.7
	Teleiophilia	98.3	98.3	100
Teleiophilia	Pedophilia	17.7	18.8	20
	Hebephilia	36	37.6	15

*Note.* Gender refers to the erotic target of interest.

Further analyses including gender preferences revealed a notable difference in overlap between sexual interests in females and overlap between sexual interest in males. Concordance indices among participants with chronophilic interests split by gender showed that 46.7% of participants with hebephilic interest in females also reported pedophilic interest in females. In contrast, 66.7% of participants with hebephilic interest in males also reported pedophilic interest in males.

### Correlations Between Pairs of Sexual Interests

As illustrated in Table 4, analysis of sexual interests regardless of gender preference showed a single significant large positive correlation between ratings of pedophilic interest and ratings of hebephilic interest (τ_b_ = .602, *p* < .001, 95% CI [.534, .663]). When split by gender of interest, a large positive correlation between ratings of pedophilic interest and ratings of hebephilic interest for females also emerged (τ_b_ = .590, *p* < .001, 95% CI [.521, .652]). Among males, there was a significant large positive correlation was found between ratings of pedophilic interest and hebephilic interest (τ_b_ = .655, *p* < .001, 95% CI [.594, .709]), and a significant small positive correlation was found between ratings of pedophilic interest and teleiophilic interest (τ_b_ = .256, *p* < .001, 95% CI [.160, .347]). Finally, a significant medium positive correlation was observed between ratings of hebephilic interest and teleiophilic interest (τ_b_ = .378, *p* < .001, 95% CI [.289, .461]). The results also indicate that the 95% confidence intervals between genders do not overlap for the pedophilia – teleiophilia pairs, and the hebephilia – teleiophilia pairs of sexual interests. However, the confidence intervals of pedophilia – hebephilia do overlap, meaning that no significant difference exist between genders for that specific pair of sexual interest.

**Table 4:** Kendall Tau-B Correlations and Associated 95% Confidence Intervals Between Pairs of Sexual Interests Split by Gender

Pairs of Sexual Interest		Kendall Tau-B					
		Gender					
		Combined		Female		Male	
		τ_b_	95% CI	τ_b_	95% CI	τ_b_	95% CI
Pedophilia	Hebephilia	.602**	[.534, .663]	.590**	[.521, .652]	.655**	[.594, .709]
	Teleiophilia	-.055	[-.154, .045]	-.018	[-.118, .082]	.256**	[.160, .347]
Hebephilia	Teleiophilia	-.034	[-.134, 0.66]	.003	[-.098, .103]	.378**	[.289, .461]

*Note.* Gender refers to the erotic target of interest.

**p < .01.

## Discussion

The study aimed to examine the polymorphism of sexual interest in children, exclusivity of sexual interest, and gender preference in the general population. In contrast to previous studies which primarily recruit their participants solely from sexual forums dedicated to sexual attractions to children (Bailey et al., 2016), the current study examined polymorphism of sexual attraction to children among a *community* sample of males. This methodological decision provides a more representative portrayal of the polymorphism of sexual interests in the general population. Accordingly, the findings add to the existing knowledge by demonstrating the existence of Seto’s hypothesis of developmental proximity within a community sample that mirrors the general population more closely than an offending sample. The findings revealed that males in our sample have concordant sexual interests for multiple age categories. Moreover, it was observed that sexual interests in children are mostly non-exclusive. Additionally, the study found that homosexual interest showed greater overlap in age groups compared to heterosexual interest. These results collectively provide partial support for the chronophilia theory (Seto, 2017).

### Polymorphism of Sexual Interests

Polymorphic sexual interest refers to the idea that individuals can have a diverse range of sexual interests that occur simultaneously across categories like gender (i.e., bisexuality, pansexuality) or age. The concordance rates in the present study provide a framework for understanding how sexual interests co-occur across age categories (i.e., hebephilia, pedophilia, and teleiophilia) in the overall sample. As predicted, concordance was observed for all combinations of age categories regardless of gender. In line with previous studies looking at sexual interest in adjacent age categories (Bailey et al., 2016; Martijn et al., 2020; Stephens et al., 2017), most participants with a hebephilic sexual interest and pedophilic sexual interest reported some level of attraction to the adjacent age group. Of particular interest to the chronophilia theory is the findings pertaining to the teleiophilic group, which show higher concordance with hebephilic (i.e., adjacent group) interest compared to pedophilic interest (i.e., non-adjacent group). These patterns of concordance lend support to a key assumption of the chronophilia theory, such that developmental proximity creates a predictable pattern in polymorphic sexual attraction. Seto and colleagues (1999) described this as a sexual response gradient, whereby individuals are expected to exhibit maximal attraction to a particular maturity status (e.g., teleiophilia), followed by descending ordered attraction to adjacent categories. In this case, pubescent children are developmentally closer to adults than are prepubescent children, thereby increasing the propensity for concordant sexual attraction.

### Exclusivity of Sexual Interests

When looking at concordance rates for pedophilia, most individuals reported a non-exclusive sexual interest for hebephilia and teleiophilia. These findings are consistent with previous studies looking at non-exclusivity of sexual attraction among men in the community (Martijn et al., 2020; Tozdan & Briken, 2015). In contrast, a study looking at 223 persons incarcerated for sexual offending against children found that the rate of non-exclusivity was 35% (Breiling et al., 2020). These results suggest that while most individuals with a pedophilic interest are non-exclusive, those who are exclusive may be more likely to offend, as indicated by the lower rates of non-exclusivity found within the offending sample. Indeed, prior research has also demonstrated that exclusivity of pedophilic interest is a strong predictor of sexual reoffending (Breiling et al., 2020; Eher et al., 2015). Thus, individuals with co-existing sexual interests for both children and adults may be at lower risk of acting on their chronophilic interest compared to individuals with exclusive interests.

### Gender Differences

We predicted that there would be similar overlap between sexual interest in males and females among the total sample, however, analyses revealed several differences in the amount of overlap in gender preferences across all age categories. Higher prevalence rates were observed for sexual interests in female children (17.6% pedophilia, 35.3% hebephilia) compared to male children (6.5% pedophilia, 7.1% hebephilia). This pattern is common in research; heterosexual interest is more common than homosexual interest (Crépault & Couture, 1980; Mundy & Cioe, 2019; Seto, 2017). The current study also found greater overlap between erotic-age preference groups among males compared to females. Subsequent analyses revealed multiple significant associations between sexual orientation and sexual interest in males. Moderate associations between pedophilia and teleiophilia ratings, as well as hebephilia and teleiophilia ratings were identified but these associations were specific to males. In line with the chronophilia theory, current results for same-sex interests revealed a stronger relationship between hebephilia and teleiophilia, compared to pedophilia and teleiophilia. Taken together, the differences in overlap and positive relationships in sexual interests based on gender preferences suggest that homosexual interests better illustrate the chronophilia theory (Seto, 2017) compared to heterosexual interests. It can be argued that homosexual erotic-age preferences are less likely to be exclusive, and more likely to be flexible, in contrast to heterosexual erotic-age preferences. Societal norms and cultural influences may potentially explain the gender differences in age attractions. Many cultures consider heterosexuality as the norm and reinforce stricter age boundaries for heterosexual relationships. In contrast, expectations and social pressures experienced by homosexual individuals may lead to greater fluidity and flexibility in their preferences. Hence, socialization may make heterosexual males adhere more rigidly to age boundaries compared to homosexual males. Another potential explanation for the flexibility of age attractions may be the availability of potential partners. The higher prevalence of heterosexual individuals in society allows heterosexual males to have access to a larger pool of potential partners compared to homosexual males. A greater availability of partners allows individuals to be more selective and rigid to their erotic-age preferences. Accordingly, the smaller pool of potential partners may lead homosexual males to demonstrate greater flexibility in age attractions.

### Limitations and Future Directions

The present study has a few limitations worth noting. First, these data were obtained using self-report measures, which have several inherent limitations. The accuracy of self-report surveys depends on several factors, one of which is willingness to disclose. Given the social stigma surrounding sexual interest in children, some individuals may be reluctant to openly report these interests due to fear of judgement or concerns over anonymity. Furthermore, the survey did not include any controls for social desirability, therefore it is possible that some participants modified their responses in accordance with social expectations and norms. Future studies should aim to include objectives measures of sexual interests such as Viewing Time (Schmidt et al., 2017) to supplement self-report tools. Sexual interest in the initial survey was scored on a 5-point Likert scale ranging from 1 (not at all interested) to 5 (extremely interested). Due to restriction of range, these sexual interest variables were recoded to represent either 1 (no interest) or 2 (some level of sexual interest). Individuals who initially scored 2 or higher on the sexual interest scale (i.e., slightly, moderately, very, or extremely) were assigned a code of 1. Thus, variance in sexual interest was likely lost. A larger sample size or different metric (e.g., reaction time rather than self-reported scores) would allow for more specific calculations of concordance based on level of interest. Future research should gather data from a larger sample and conduct a fine-grained analysis of the degree of overlap in the magnitude of sexual interests. Furthermore, our sample was primarily recruited from a Canadian university psychology participant pool and online sexual forums on Reddit. These recruitment methods introduce inherent biases. University participant pools tend to be younger, more educated, and more homogenous than general population, often described as “WEIRD” samples (Western, Educated, Industrialized, Rich, and Democratic). As such, this demographic does not accurately reflect the diversity of the broader population. Furthermore, participants from Reddit were recruited from online sexual forums focusing on discussions pertaining to sex education and advice, which also does not represent the broader population. Moreover, although the SLSB scale utilized in this study has a demonstrated history of comprehensively assessing paraphilias in non-offender samples, there has been a lack of studies to support its psychometric validity and reliability. Finally, given that our sample was recruited in part via internet forums, it may not be fully representative of the population, which is a common limitation in this area of research.

### Clinical Implications

The present study found a high degree of polymorphic sexual interest in the overall community sample. Most individuals who reported sexual attraction to children also reported concordant sexual attraction to adults, which may present an important avenue for clinical intervention. For example, Tozdan and Briken (2017) found that individuals who reported more flexibility in their sexual preferences were more motivated to alter their sexual interests. Individuals with concordant sexual interest in both children and adults may have more opportunities for consensual adult sexual relationships compared to individuals with exclusive interest in children. Having legal alternative outlets for sexual gratification may therefore prove to be an important factor in therapeutic success. However, while polymorphic sexual attraction is a marker of concordant sexual preference, it does not completely negate the concept of sexual preference. As shown in the community sample used in present study, the large majority of individuals reporting sexual attraction to multiple age categories simultaneously exhibit a higher degree of preference for one age group over another. Research evaluating polymorphic sexual interest should consider not only the presence of simultaneous sexual interests, but the respective strength of those interests. Understanding sexual attractions in non-offending populations may provide critical insight into protective factors that prevent the manifestation of harmful behaviours. Future research may be able to uncover mechanisms that lend well to impulse control and the adherence to legal boundaries.

## Data Availability

The data supporting the findings of this study can be made available upon request.
